# A Bibliometric Analysis of Reactive Oxygen Species Based Nanotechnology for Cardiovascular Diseases

**DOI:** 10.3389/fcvm.2022.940769

**Published:** 2022-07-05

**Authors:** Yun Liang, Shenjie Liao, Xiaoshen Zhang

**Affiliations:** ^1^Department of Cardiovascular Surgery, The First Affiliated Hospital, Jinan University, Guangzhou, China; ^2^Academician Workstation, Affiliated Hospital of North Sichuan Medical College, Nanchong, China

**Keywords:** nanotechnology, reactive oxygen species, bibliometric analysis, cardiovascular disease, CVD risk assessment, CVD risk prediction, data visualization analysis

## Abstract

Cardiovascular diseases (CVDs) continue to be the leading cause of health problems around the world. Because of its unique properties, reactive oxygen species (ROS)-based nanotechnology offers novel solutions to the diagnosis and treatment of CVDs. In order to identify and further promote the development of ROS-based nanotechnology in CVDs, we here provide a bibliometric analysis. 701 eligible articles about the ROS–based nanotechnology for CVD up to May 26th, 2022, were taken from the Web of Science Core Collection database. The VOSviewer was used to analyze annual publications, countries/institutions, funding agencies, journals and research category, and the research hotspots. From the publication of the first article in 2005 to 2021, the output and the number of citations of articles are on the rise. Based on the bibliometric analysis, we found that the current research focuses on the correlation between diagnosis (sensors and), treatment (oxidative stress, inflammation, and drug delivery) and safety (toxicity). Since 2019, research on nanomedicine and drug delivery has become a hotspot. So, more research in chemistry, materials, biology, and medicine is required to further develop and construct ROS-based nanomaterials.

## Introduction

According to World Health Organization (WHO) estimates, cardiovascular diseases (CVDs) are one of the most important causes affecting health status and the leading cause of death worldwide (1).

Cardiovascular diseases are a broad term for any condition that affects the heart or blood vessels, such as high blood pressure, stroke, coronary heart disease, myocardial infarction, etc (2). However, accumulating evidences demonstrated that some patients’ CVDs may due to oxidative stress injury caused by excessive production of reactive oxygen species (ROS) (3). ROS are a group of highly reactive molecules and free radicals that are made by the metabolism of molecular oxygen (4). Under normal circumstances, ROS are produced at low levels in all cells *via* the electron-transport chain during aerobic respiration and by a variety of constitutively active oxidases (5). However, in the presence of CVDs, the excessive production of ROS that cannot be neutralized by antioxidants in cells [such as superoxide dismutase (SOD) and catalase] and results in oxidative stress may aggravate myocardial damage and impair cell components such as membrane lipids, proteins, and DNA (6). In other words, this also provides an efficient approach for dealing with and preventing CVDs *via* the regulation of ROS. However, current medical methods, leading to ineffective or excessive treatment, cannot take advantage of the changes in ROS concentration in the microenvironment of diseased tissues (7). Nanotechnology has the potential to bring up new avenues for assisting or overcoming constraints in CVD diagnosis and treatment due to its unique features. ROS–based nanotechnology focuses on the ROS–related nano chemistry interface (ROS generation, ROS transition, and ROS scavenging) for improved reaction efficiencies, as well as the ROS-related nanobiology interactions (bioavailability, biodegradability, and biocompatibility) for improved therapeutic outcomes. In recent years, various advanced ROS-based nanotechnologies have been developed and applied to the diagnosis and treatment of cardiovascular illnesses in response to the growing interest of scholars (8). Inspired by the vast advances in the field of ROS-based nanotechnology for CVD, we present this article regarding ROS-based nanotechnology for CVD *via* the aid of bibliometric analysis, with the aim of identifying current research advances and further promoting the development of this important field. In this work, the Web of Science(WoS) innate function and bibliometric methods are used to identify annual publication trends, the most productive countries/institutions, publication-related journals, and research hotspots in the field. Based on the macroscopic and objective results obtained from this analysis, we further proposed a prospects for future research directions and clinical translations of the applications of ROS-base nanotechnology for CVDs. This paper may serve as a reference, not only for researchers, but also for doctors who work in the field of nanotechnology in CVD.

## Materials and Methods

All publications relating to ROS–based nanotechnology for CVD were retrieved from Clarivate analysis’s Web of Science Core collection Database including editions of SCI-EXPANDED (2003–present), SSCI (2003–present), A & HCI (2003–present), ESCI (2015–present), CCR–EXPANDED (1985–present), and IC (1993–present) (9, 10). The data was obtained on May 26th, 2022 and the following search query was developed to extract the total number of published items.

TS = (nanoparticle* or nanomaterial* or nanodot* or “quantum dot*” or nanosphere* or nanorod* or nanofiber* or nanotube* or nanosheet* or nanocomposite* or nanodevice* or nanocluster* or nanotech* or nanocarrier* or nanowire* or nanoliposome* or nanoemulsion* or nanocrystal* or nanogels* or nanoconjugate* or nanodiamond*) (11) and TS = (“high blood pressure” or hypertension* or “peripheral arter*” disease* or “atrial fibrillar*” or tachycardia* or endocarditis* or pericard* or ischem* or arrhythmia* or thrombo* or cardio* or cardiac* or “heart failure” or “heart beat” or “heart rate*” or “heart val*” or coronary* or angina* or ventric* or myocard*) (11) and TS = (“reactive oxygen species” or “ROS” or “H2O2” or “hydrogen peroxide”) (12).

Additional, the language was limited to “English.” We extracted the following information about the studies: year of publication, distribution of articles by countries or institutions, funding agencies, authors, keywords, and major journals’ published articles. VOSviewer was employed to conduct the data visualization.

## Results

### Search Results

A total of 713 publications were retrieved (Supplementary Figure 1). These included 616 articles, 85 reviews, 6 meeting abstracts and other types of papers. Document types such as corrections, meeting abstracts, and editorial materials do not share the same citation rates, therefore, only document types including original articles and reviews were utilized (in total 701 publications) in our following analysis (Supplementary Table 1).

### The Annual Trend of Paper Publication Quantity

**FIGURE 1:**
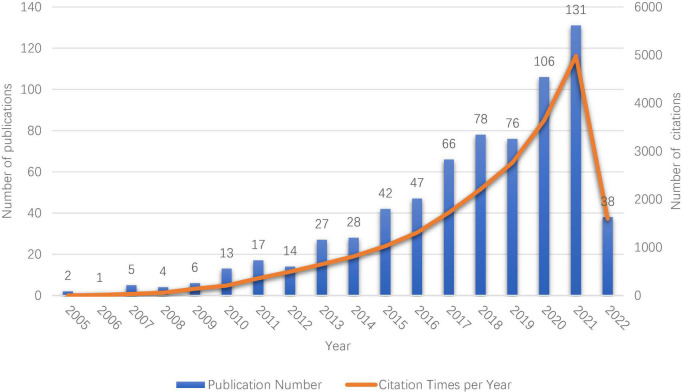
The annual publication and citation growth of literature.

Figure 1 illustrates the annual publications and citations growth of literatures regarding ROS–based nanotechnology for CVD. It displayed an upward trend from 2 documents published in 2005 to 131 documents published in 2021. The period from 2013 to 2021 was a rapid development period. Citation analysis is a simple and objective way to evaluate the quality of published articles, journals, research organizations and even individual researchers; The times of an articles cited reflect its scientific impact (13). Among all the retrieved articles, there were 18,474 citing documents including 279 self-citations and 18,195 without self-citation (accounted for 98.49% of all citing documents). Sum of the times cited was 22,032 including 21,360 without self-cited which accounted for 96.95%.

### Most Productive Countries/Regions and Institutions

**FIGURE 2:**
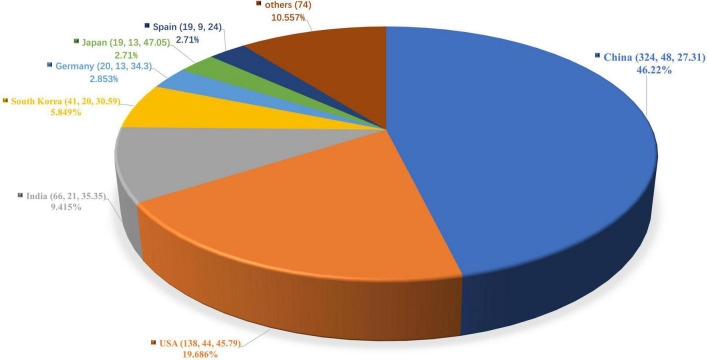
Distribution of publications in countries/regions. The number in the brackets goes: (publication numbers, h-index, average citations). Percentage indicates the proportion of the publication number in global scale.

**FIGURE 3:**
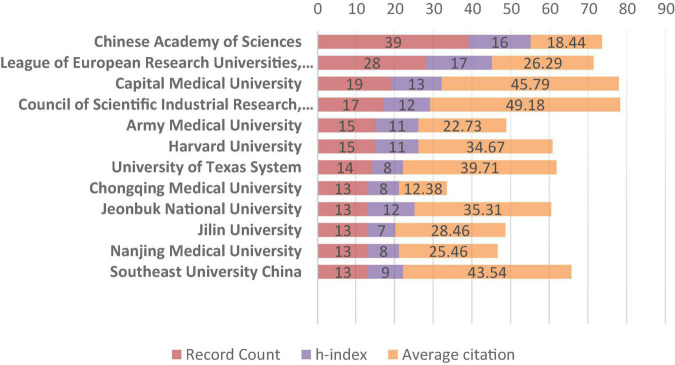
The most contributing institutes in the field of ROS based nanotechnology for cardiovascular diseases.

Of these 701 documents, 60 countries and regions contributed to this research field. China was the most productive country which published the maximum number of related studies (*n* = 324, accounted for 41.45% of all). It was followed by the United States (*n* = 138, 19.686%), India (*n* = 66, 9.415%), South Korea (*n* = 41, 5.849%) and Germany (*n* = 20, 2.853%). In addition, the average citations and h-index of the top 7 most productive and influential countries are also presented (Figure 2). With this, China, the United States, and India rank in top three positions in terms of the h-index, reflecting a relatively high number of publications and associated citations from these countries. Japan topped the list with the highest average citations (47.05), followed by the United States (45.79) and India (35.35). When it comes to institution rankings (Figure 3), the Chinese Academy of Sciences takes the first rank position in terms of publication numbers, with 39 publications, while League of European Research Universities occupies the leading institution in terms of the h-index (14). Furthermore, the Council of Scientific Industrial Research ranked the first position in terms of the average citations (49.18).

### Productivity by Funding Agencies

**FIGURE 4:**
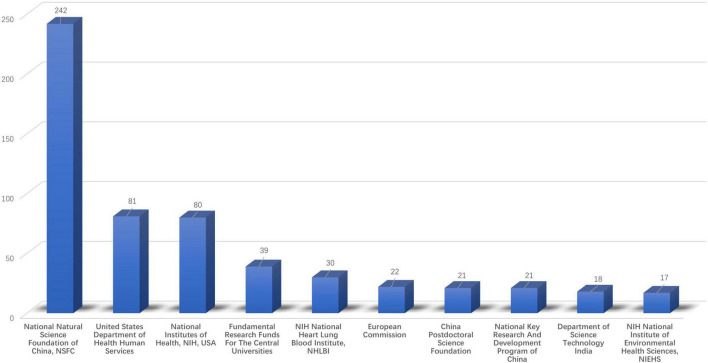
Distribution of articles by funding agencies (top 10 funding agencies).

Based on the selection criteria, the top 10 funding agencies are represented in Figure 4. Among these funding agencies, the National Natural Science Foundation of China (NSFC) ranked first, accounting for 242 funded projects. The United States Department of Health Human Services and National Institutes of Health in the United States ranked second and third, with 81 funded projects and 80 funded projects, respectively. However, 100 articles, which accounted for nearly 14.265% of the total, were without any funding support.

### Journals and Research Category Analysis

**FIGURE 5:**
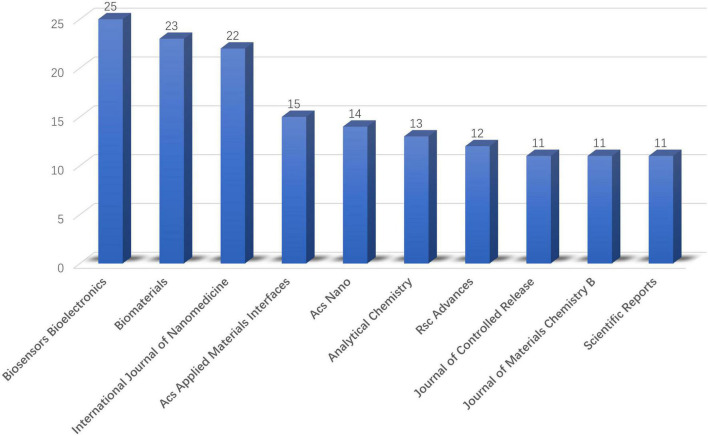
Productivity of journals (top 10 journals).

These 701 documents were published in 296 kinds of journals. The top 10 most productive journals in this area were shown on Figure 5. These top 10 journals published a 157 total of documents, accounting for 22.40% of all. Biosensors Bioelectronics published the most document (*n* = 25). The second most popular journal was Biomaterials which has 23 publications. International Journal of Nanomedicine ranked No. 3 (*n* = 22) and was followed by ACS Applied Materials Interfaces (*n* = 15) and ACS Nano (*n* = 14).

**FIGURE 6:**
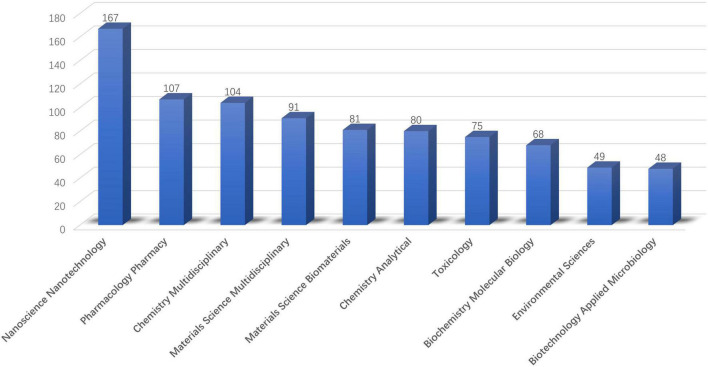
The top 10 research categories.

In terms of research categories, a total of 74 research categories were involved. Based on the quantity of publications, the top 10 research categories are demonstrated in Figure 6. “Nanoscience Nanotechnology” (167 publications), “Pharmacology Pharmacy” (107 publications), “Chemistry Multidisciplinary” (104 publications), “Materials Science Multidisciplinary” (91 publications) and “Materials Science Biomaterials” (81 publications) are ranked in top 5 positions. As for the publisher, the most represented publishers based on the number of the publications were “Elsevier” (225 publications), “Springer Nature” (80 publications), “Amer Chemical Soc” (74 publications), “Wiley” (58 publications), and “Royal Soc Chemistry” (45 publications).

### Co-citation Analysis of Cited Journals

**FIGURE 7:**
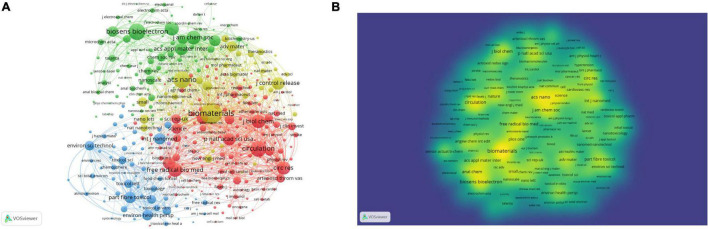
Co-citation analysis of citated sources. Node size suggests the citation amount. Settings in VOSviewer Analysis:Minimum number of citations of a source: 20 (of the 4,772 sources, 378 meet the threshold) Method: Association strength; Layout: Attractions 6, Repulsion −1. Clustering: Resolution 1.00, Min.cluster.size 1. **(A)** Network mapping of citated sources (four clusters). **(B)** Density mapping according to the citation amount of a source (yellow means more of a source).

A co-citation analysis of cited journals identifies the most relevant sources cited by publications in the specified topic (15). This analysis can identify the leading journals in the field of CVD that cover ROS-based nanotechnology. In addition, it can also offer information with regard to research topics in the field of ROS–based nanotechnology in CVD, as indicated by journal titles. With an analysis by means of the VOSviewer, 4,772 cited journals were referred by the 701 publications. 378 journals with employed the threshold at 20 citations were used for co-citation analysis and presented in Figure 7. The top 5 cited journals are Biomaterials (994 citations), ACS nano (687 citations), Biosensors Bioelectronics (662 citations), Circulation (617 citations) and Anal Chem (494 citations). Most of the journals are associated with nanoscience while cardiovascular-focused journals such as Circulation, J Clin Invest, Circ Res, J Am Coll Cardiol or Cardiovasc Res. were also recognized in our database. As can be inferred from the clusters, the major fields of ROS-based nanotechnology in CVD application lie in biomedicine (red color)-, biosensor (green)-, toxicology (blue)- and biomaterials/nanomaterials (yellow)- related science.

### Research Hotspots in Reactive Oxygen Species–Based Nanotechnology for Cardiovascular Diseases Applications

**FIGURE 8:**
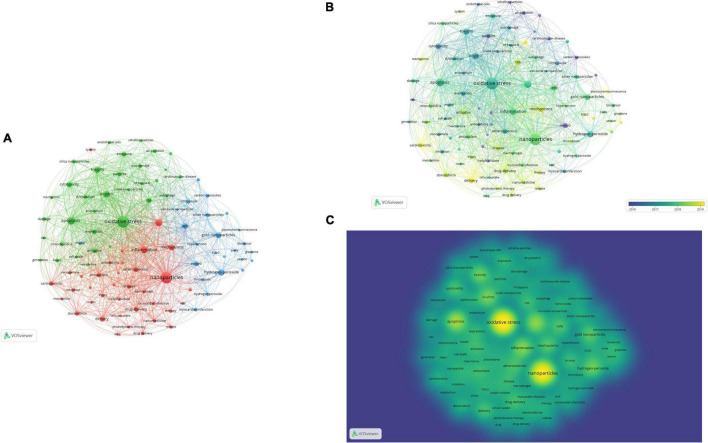
Co-occurrence analysis of all keywords with an occurrence frequency more than 12 (of the 3,922 keywords, 93 meet the threshold). Colors are used to indicate clusters. Circle size is based on the number of occurrence. Settings in VOSviewer Analysis: Method: Lin Log/modularity; Resolution: 1.0; Minimum cluster size: 2; Attractions:1; Repulsion:-1. **(A)** Network mapping of keywords of related studies (three clusters). **(B)** Overlay mapping of keywords according to average publication year (blue means earlier, yellow means later). **(C)** Density mapping of keywords according to the frequency of appearance (yellow means more frequent).

In order to obtain a general understanding of the research focuses of the ROS–based nanotechnology in CVD field, a co- occurrence analysis of keywords was conducted by using VOS viewer (Figure 8). A keyword co-occurrence landscape of 93 keywords, acquired from 701 publications, with a minimum occurrence of 12 times, is presented. The top 10 most frequently occurring keywords are oxidative stress (occurred 237 times), nanoparticles (215 times), apoptosis (101 times), ROS (85 times), inflammation (80 times), *in vitro* (72 times), toxicity (67 times), hydrogen-peroxide (65 times), mechanisms (58 times), and gold nanoparticles (56 times). Such high-frequency keywords indicate that the major application of ROS–based nanotechnology in CVD lies in oxidative stress, apoptosis, inflammation, mechanisms and other theragnostic-related fields. In addition, three clusters in different colors indicating different research fields were identified and adjusted by the innate function of the VOSviewer. As indicated, the largest red cluster, which is related to the regulation of oxidative stress, was the most significant application of ROS–based nanotechnology in CVD. The second biggest cluster was colored in green, demonstrating the mechanism and toxicity of nanomaterials for CVD. In addition, the third biggest cluster was colored in blue, demonstrating the construction and functional verification of novel nano materials based on ROS. Likewise, it can be inferred from this result that ROS–based nanotechnology in CVD is targeted mainly at diagnosis (sensor and), therapy (oxidative stress, inflammatory and drug delivery) and safety (toxicity) studies. Meanwhile, through the analysis of Figure 8B, we also see that since 2019, the research on nanomedicine and drug delivery has become a hotspot.

## Discussion

With the help of bibliometric analysis, this paper was given about ROS-based nanotechnology for CVDs. Up to May 26th, 2020, the total number of documents on this topic was 713. Finally, only 701 documents, including original articles and reviews, were used for bibliometric analysis. The publication output was rich in the period from 2013 to 2021 and displayed an upward trend. It is explicit that research on ROS-based nanotechnology for CVD has advanced rapidly over the last decade. ROS-based nanotechnology for CVD is predicted to remain a research hotspot in the future. Among all the retrieved 701 articles, a total of 22,032 citations was received with an average time cited per document of 31.43. It was seen that the number of citations has increased 4983-fold since 2005 to 2021, reflecting the rising importance of this research area.

In total, 41.45% of all these articles were from China, and the Chinese Academy of Sciences takes the first rank position in terms of publication numbers. Through analysis, we note that China was also the leading country with the maximum average citations. Additionally, India ranks third in the world in terms of article output and h-index. In terms of institutional contribution, the Chinese Academy of Sciences ranks first with the highest document output. However, the League of European Research Universities in the United States leads in terms of the h-index, while the Council of Scientific Industrial Research in India leads in terms of average citations. The achievements of two developing countries with large populations (China and India) in this field have greatly encouraged the innovation of global CVD prevention and treatment technology.

Based on the funding source for these articles, we found that China and the United States have the most funded projects in this field. Accordingly, it also explains why China and the United States have made great achievements in this field. We speculate that nanotechnology has become a priority for government funding in the two countries as a means of safeguarding healthcare. It is admirable that Indian scholars have received relatively few fund-supported projects, but their literature output and quality are high. However, about 14.265% of the articles were not funded by any funding agency. It is clear that the significance of ROS-based nanotechnology for CVD is still underappreciated in many countries.

Among the top 10 most productive journals out of 296 kinds of journal involved, 7 of them were categorized in “Chemistry” or “Materials,” while the remaining 3 were, respectively, listed in “medicine” (*n* = 2), and “Comprehensive” (*n* = 1). Of the top 10 research categories, 8 categories belong to the categories of “chemistry or materials science,” while 2 categories belong to the categories of “medicine.” From this analysis result, we can infer that the application of ROS based nanotechnology in the field of CVD is more reflected in innovation in the field of chemistry and materials.

The top 5 journals with the largest number of articles accounted for 68.76% of all the publications. Among them, journals belonging to Elsevier publishers accounted for 32.097%. “Amer chemical SOC” and “Royal SOC chemistry” are the top journals in the professional field, and the number of papers included accounts for 16.98% of the total. Through further co-citation analysis of cited journals, we found that the application of ROS based nanotechnology in CVD mainly focuses on biomedicine, biosensors, toxicology, and biomaterials/nanomaterials related science. So far, the analysis results have proved that nanotechnology combines materials science, chemistry, and biology and is involved in the diagnosis and treatment of CVDs by sensing and regulating ROS levels (16).

**FIGURE 9:**
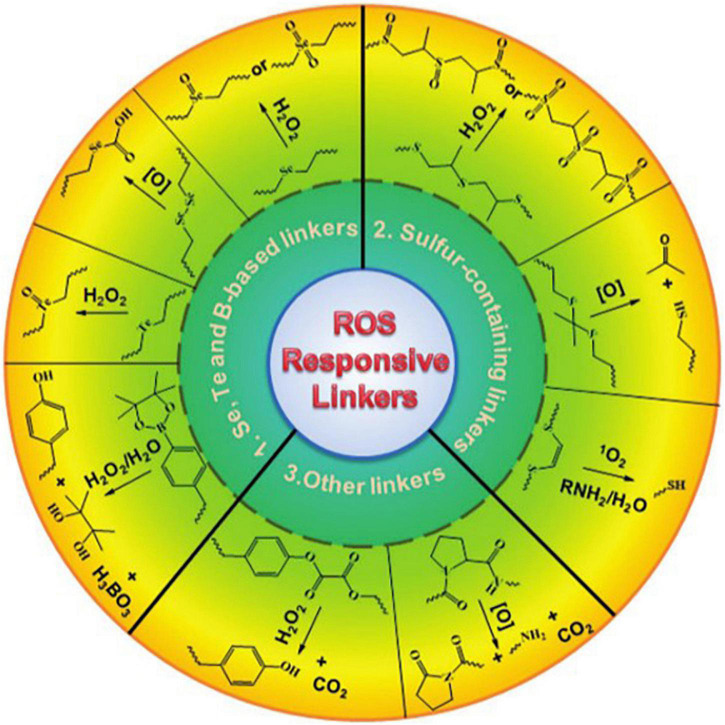
Representative ROS-responsive linkers that have employed for the design of ROS-responsive drug carriers. Printed with permission from Willey (19). Copyright 2016.

Then, using keyword co-occurrence analysis, the research focuses of ROS-based nanotechnology in the CVD field were determined. The result shows that ROS-based nanotechnology in CVD is targeted mainly at diagnosis (sensor and), therapy (oxidative stress, inflammatory, and drug delivery) and safety (toxicity) studies. Additionally, the research on nanomedicine and drug delivery has become a hotspot since 2019. Current studies have demonstrated that oxidative stress is the key mechanisms of CVDs (17). As one of the application means of ROS-based nanotechnology, the drug delivery system acts on cardiovascular lesions by targeting, thus more effectively reducing oxidative damage and eliminating inflammation (18). This abnormal biochemical change in CVDs has prompted researchers to use unbalanced ROS levels to develop target-specific drug delivery systems (14). ROS-based drug delivery and nanomedicine will primarily focus on polymeric nanoparticles, hydrogels, inorganic nanoparticles, and activatable prodrugs integrated with diverse ROS-responsive moieties for spatiotemporally controlled drug release for effective therapy (19). The design goal of ROS-based nanomaterial is to match the degradation rate and dynamic ROS concentration of the material itself, which is very important for timely detection of signal molecules, releasing drug loading as needed, and regulating the growth and growth of transfer cells (20). By utilizing ROS-responsive materials and linkers (Figure 9), various ROS-responsive drug delivery systems have been developed and investigated for therapeutic purposes. So, more research in chemistry, materials, biology, and medicine is required to further develop and construct ROS-based nanomaterials (21).

## Conclusion

China, the United States, and India, 60 countries or regions dominated by these three countries, have made outstanding contributions to research in the field of ROS based nanotechnology applied to CVDs. From the publication of the first article in 2005 to 2021, the output and the number of citations of articles are on the rise. The current research focuses on the correlation between diagnosis (sensors and), treatment (oxidative stress, inflammation, and drug delivery) and safety (toxicity). Since 2019, research on nanomedicine and drug delivery has become a hotspot. To further develop and construct ROS-based nanomaterials, more research in chemistry, materials, biology, and medicine is required.

## Data Availability Statement

The original contributions presented in this study are included in the article/Supplementary Material, further inquiries can be directed to the corresponding author.

## Ethics Statement

The sample data in this manuscript comes from the secondary processing of the existing data and does not involve the ethical issues of animals and human beings.

## Author Contributions

YL: formal analysis, data curation, and writing – original draft. SL: data curation. XZ: supervision and writing – review and editing. All authors contributed to manuscript revision, read, and approved the submitted version.

## Conflict of Interest

The authors declare that the research was conducted in the absence of any commercial or financial relationships that could be construed as a potential conflict of interest.

## Publisher’s Note

All claims expressed in this article are solely those of the authors and do not necessarily represent those of their affiliated organizations, or those of the publisher, the editors and the reviewers. Any product that may be evaluated in this article, or claim that may be made by its manufacturer, is not guaranteed or endorsed by the publisher.

## Supplementary Material

Supplementary Figure 1 The retrieval strategy and results of publications. The search was conducted in the WoS core collection database on May 26th, 2022.

Supplementary Table 1 The 701 analyzed papers.

## References

[1] Nowbar AN Gitto M Howard JP Francis DP Al-Lamee R. Mortality from ischemic heart disease: analysis of data from the world health organization and coronary artery disease risk factors from NCD risk factor collaboration. *Circ Cardiovasc Qual Outcomes.* (2019) 12:1–11. 10.1161/CIRCOUTCOMES.118.005375 PMC6613716 31163980

[2] Zhao D Liu J Wang M Zhang X Zhou M. Epidemiology of cardiovascular disease in China: current features and implications. *Nat Rev Cardiol.* (2019) 16:203–12. 10.1038/s41569-018-0119-4 30467329

[3] Zhao T Wu W Sui L Huang Q Nan Y Liu J Reactive oxygen species-based nanomaterials for the treatment of myocardial ischemia reperfusion injuries. *Bioact Mater.* (2022) 7:47–72. 10.1016/j.bioactmat.2021.06.006 34466716 PMC8377441

[4] Li R Jia Z Trush MA. Defining ROS in biology and medicine. *React Oxyg Species (Apex).* (2016) 1:9. 10.20455/ros.2016.803 29707643 PMC5921829

[5] Xu Q He C Xiao C Chen X. Reactive oxygen species (ROS) responsive polymers for biomedical applications. *Macromol Biosci.* (2016) 16:635–46. 10.1002/mabi.201500440 26891447

[6] Joshi-Barr S de Gracia Lux C Mahmoud E Almutairi A. Exploiting oxidative microenvironments in the body as triggers for drug delivery systems. *Antioxid Redox Signal.* (2013) 21:730–54. 10.1089/ars.2013.5754 24328819 PMC4098119

[7] Chen W Li D. Reactive oxygen species (ROS)-responsive nanomedicine for solving ischemia-reperfusion injury. *Front Chem.* (2020) 8:732. 10.3389/fchem.2020.00732 32974285 PMC7472733

[8] Sabir F Barani M Mukhtar M Rahdar A Cucchiarini M Zafar MN Nanodiagnosis and nanotreatment of cardiovascular diseases: an overview. *Chemosensors.* (2021) 9:67.

[9] Ahmad T Murad MA Baig M Hui J. Research trends in COVID-19 vaccine: a bibliometric analysis. *Hum Vaccines Immunother.* (2021) 17:2367–72. 10.1080/21645515.2021.1886806 33687303 PMC8475596

[10] Khan M Ahmad T Khan MM Murad MA Baig M Ali A Research trends in polio during the last 50 years: a bibliometric analysis. *J Univ Med Dent Coll.* (2022) 13:341–6.

[11] Zheng Z Zhu S Lv M Gu Z Hu H. Harnessing nanotechnology for cardiovascular disease applications - a comprehensive review based on bibliometric analysis. *Nano Today.* (2022) 44:101453. 10.1016/j.nantod.2022.101453

[12] Zhang Z Dalan R Hu Z Wang J Chew NWS Poh K Reactive oxygen species scavenging nanomedicine for the treatment of ischemic heart disease. *Adv Mater.* (2022) 8:2202169. 10.1002/adma.202202169 35470476

[13] Sevinc A. Web of science: a unique method of cifed reference searching. *J Natl Med Assoc.* (2004) 96:980–3. 15253331 PMC2568431

[14] Yao Y Zhang H Wang Z Ding J Wang S Huang B Reactive oxygen species (ROS)-responsive biomaterials mediate tissue microenvironments and tissue regeneration. *J Mater Chem B.* (2019) 7:5019–37. 10.1039/c9tb00847k 31432870

[15] Liu Y Zhu S Gu Z Zhao Y. A bibliometric analysis: research progress and prospects on transition metal dichalcogenides in the biomedical field. *Chin Chem Lett.* (2021) 32:3762–70.

[16] Purcell BP Lobb D Charati MB Dorsey SM Wade RJ Zellars KN Injectable and bioresponsive hydrogels for on-demand matrix metalloproteinase inhibition. *Nat Mater.* (2014) 13:653–61. 10.1038/nmat3922 24681647 PMC4031269

[17] González-Montero J Brito R Gajardo AI Rodrigo R. Myocardial reperfusion injury and oxidative stress: therapeutic opportunities. *World J Cardiol.* (2018) 10:74–86. 10.4330/wjc.v10.i9.74 30344955 PMC6189069

[18] Hajipour MJ Mehrani M Abbasi SH Amin A Kassaian SE Garbern JC Nanoscale technologies for prevention and treatment of heart failure: challenges and opportunities. *Chem Rev.* (2019) 119:11352–90. 10.1021/acs.chemrev.8b00323 31490059 PMC7003249

[19] Saravanakumar G Kim J Kim WJ. Reactive-oxygen-species-responsive drug delivery systems: promises and challenges. *Adv Sci.* (2017) 4:1600124. 10.1002/advs.201600124 28105390 PMC5238745

[20] Agrahari V Agrahari V. Facilitating the translation of nanomedicines to a clinical product: challenges and opportunities. *Drug Discov Today.* (2018) 23:974–91. 10.1016/j.drudis.2018.01.047 29406263

[21] Pelaz B Alexiou C Alvarez-Puebla RA Alves F Andrews AM Ashraf S Diverse applications of nanomedicine. *ACS Nano.* (2017) 11:2313–81. 28290206 10.1021/acsnano.6b06040 PMC5371978

