# Supplementary figures and images for: A Bibliometric Analysis of Reactive Oxygen Species Based Nanotechnology for Cardiovascular Diseases
Source: Front Cardiovasc Med. 2022 Jul 5;9:940769. doi: 10.3389/fcvm.2022.940769 (PMC9294284; doi:10.3389/fcvm.2022.940769)

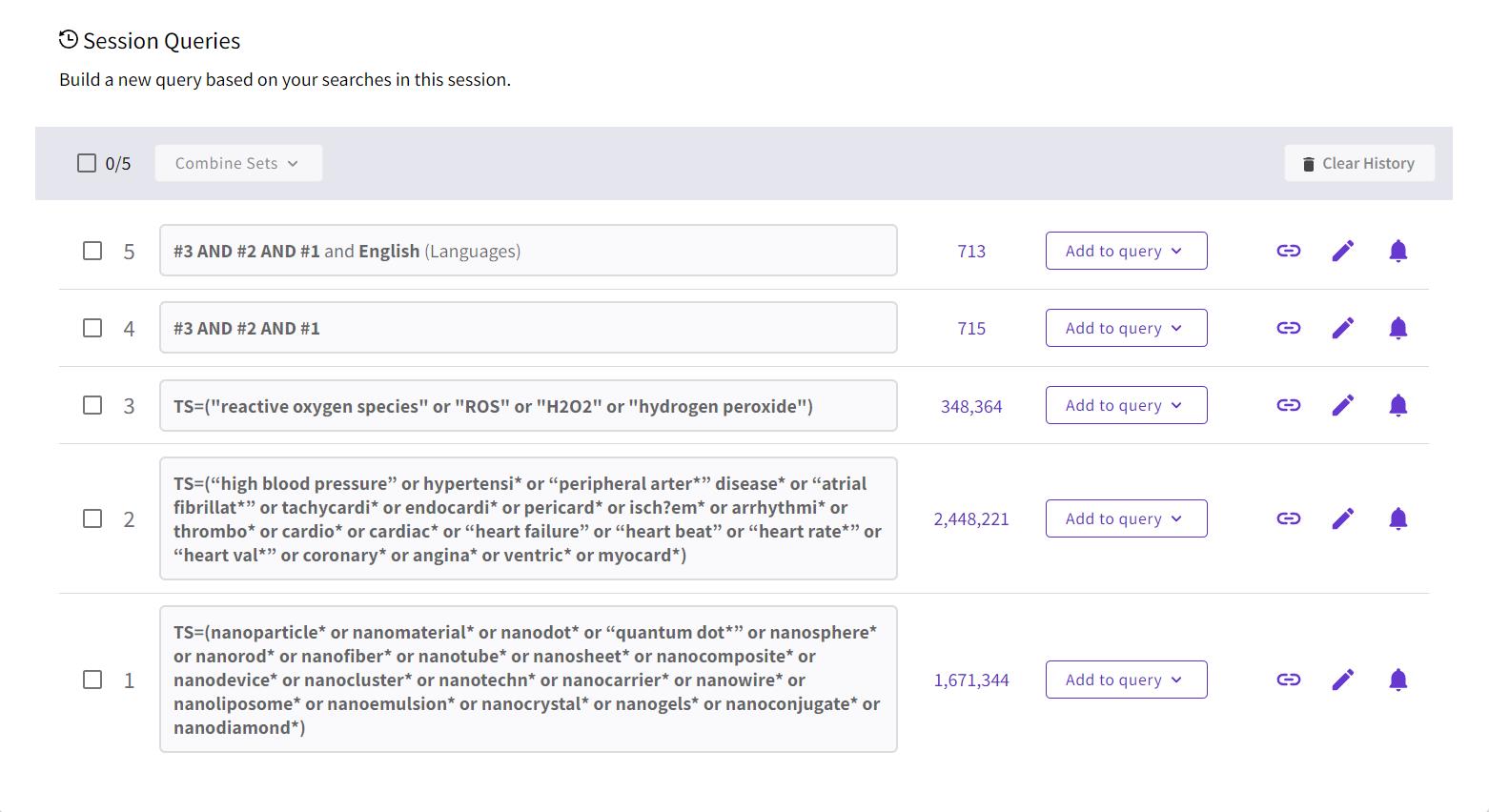

Supplement: Supplementary Figure 1 — The retrieval strategy and results of publications. The search was conducted in the WoS core collection database on May 26th, 2022. [file Image_1.JPEG]
